# Novel Microsatellite Markers Used for Determining Genetic Diversity and Tracing of Wild and Farmed Populations of the Amazonian Giant Fish *Arapaima gigas*

**DOI:** 10.3390/genes12091324

**Published:** 2021-08-27

**Authors:** Paola Fabiana Fazzi-Gomes, Jonas da Paz Aguiar, Diego Marques, Gleyce Fonseca Cabral, Fabiano Cordeiro Moreira, Marilia Danyelle Nunes Rodrigues, Caio Santos Silva, Igor Hamoy, Sidney Santos

**Affiliations:** 1 Laboratório de Humana e Médica, Universidade Federal do Pará, Rua Augusto Correa, 1, Belém 66075-110, Brazil; paolafazzi.gomes@yahoo.com.br (P.F.F.-G.); diegomarquescs@outlook.com (D.M.); cabralffg@gmail.com (G.F.C.); fabiano.ufpa@gmail.com (F.C.M.); scaio@hotmail.com (C.S.S.); 2 Universidade Federal do Pará, Campus Bragança, Alameda Leandro Ribeiro s/n, Bragança 68600-000, Brazil; jonaspaguiar@gmail.com; 3 Laboratório de Genética Aplicada, Instituto de Recursos Aquáticos e Socioambientais, Universidade Federal Rural da Amazônia, Avenida Presidente Tancredo Neves, 2501, Belem 66077-830, Brazil; nunes.mdnunes@gmail.com (M.D.N.R.); ighamoy@gmail.com (I.H.)

**Keywords:** genetic tracing, *Arapaima gigas*, microsatellites, aquaculture, fishing management, Amazon, genetic diversity

## Abstract

The Amazonian symbol fish *Arapaima gigas* is the only living representative of the Arapamidae family. Environmental pressures and illegal fishing threaten the species’ survival. To protect wild populations, a national regulation must be developed for the management of *A. gigas* throughout the Amazon basin. Moreover, the reproductive genetic management and recruitment of additional founders by aquaculture farms are needed to mitigate the damage caused by domestication. To contribute to the sustainable development, we investigated the genetic diversity of wild and cultivated populations of *A. gigas* and developed a panel composed by 12 microsatellite markers for individual and population genetic tracing. We analyzed 368 samples from three wild and four farmed populations. The results revealed low rates of genetic diversity in all populations, loss of genetic diversity and high inbreeding rates in farmed populations, and genetic structuring among wild and farmed populations. Genetic tracing using the 12 microsatellite markers was effective, and presented a better performance in identifying samples at the population level. The 12-microsatellite panel is appliable to the legal aspects of the trade of the *A. gigas*, such as origin discrimination, reproductive genetic management by DNA profiling, and evaluation and monitoring of genetic diversity.

## 1. Introduction

The Amazon symbol fish *Arapaima gigas* (Schinz, 1822) is the only living representative of the Arapamidae family. It is commonly referred to as pirarucu, a name that comes from two indigenous native terms, *pira* (which means fish) and *urucum* (which is a red-colored seasoning called annatto), because of the species’ red tail color [1]. 

The species was given the title of “Amazonian’s codfish”, and features such as its large size (weighing up to 200 kg) and excellent flavored meat make the *A. gigas*’ marketing extremely profitable, compared with other species. Additionally, it has great cultural and economic value for riverside communities that survive from the *A. gigas*’ fishing [2].

The *A. gigas*’ marketing is mostly supplied by specimens coming from fishing, and the largest fishing landing harbor is located in the state of Amazonas, with production coming from federal and state environmental conservation units, indigenous lands and regions with current fishing agreements due to predatory fishing and risk of extinction of the species. Another smaller portion of *A. gigas*’ commercial trade originates from farming, in various aquaculture production systems.

According to the last farming census, 3.246 tons of *A. gigas* meat was produced, which represents 0.43% of all aquaculture production in Brazil [3]. Despite the low production, the species is indicated among the native fish with the greatest potential for aquaculture because of its high growth (reaching 10 kg in one year of cultivation), high fillet yield and its rusticity [4]. 

In recent years, commercial sales to consumers of *A. gigas* products and by-products have broadened in Brazil and abroad. The fillet of *A. gigas*’ meat is already in high demand by the international gourmet market [5]. Additionally, its leather has a high market value for the confection of accessories, such as bags and shoes, and it will likely exceed meat exports in the next years.

In a recent study [6], there is warning of the risk of new threats to wild stocks of *A. gigas*, due to the increase in commercial activity to mainly serve the rising leather market. Data from the United States Law Enforcement Management Information System (LEMIS) show that, among the incidents involving arapaima leather products, 89% were originated from wild-caught animals, and they all originated from Brazil [6].

It is important to emphasize that wild stocks of *A. gigas* in Brazil were on the verge of collapse in the 60s and 70s due to overfishing; consequently, part of the genetic diversity has been lost. Despite positive signs of recovery in terms of population size in managed areas, there are still data deficits regarding the diversity and genetic structure of wild populations of *A. gigas*—until now there is also an absence of these data for the farmed ones. The genetic diversity is the starting point of adaptation, evolution, and survival, without which the population is more susceptible to extinction [7]. In farming conditions, it is the source of genetic variation for future breeding programs [8], thus the knowledge of the genetic diversity is essential for the sustainability of species. 

Nowadays, one of the main threats to the wild populations of *A. gigas* is the illegal trade, by which animals are caught in prohibited areas and traded illegally or, even, with an indication that they come from a farming environment [9]. The Convention on International Trade in Endangered Species of Wild Fauna and Flora—CITES—recommends the use of forensic genetics in surveillance practices as a gold standard against this issue, since genetic evidence has a crucial advantage over other types of documentaries, tangible, physical or biological evidence because it confers accurate confirmation for identifying or tracing [10]. 

This process is based on the identification of individuals and populations by genetic tracing. At the individual level, the process is based on parental screening for the identification of individuals. At the population level, it is based on identifying allelic frequencies using probabilistic or deterministic approaches [11]. For the *A. gigas*, the genetic tracing can be useful against the illegal trade through genetic proof of the origin of the marketed animals, and it could also help in the implantation of breeding genetic management in the aquaculture farms based on the identification of DNA profiles. 

In studies of diversity and genetic tracing of aquatic species, microsatellite molecular markers are the most used genomic markers, mainly due to their high polymorphism rates, their abundance throughout the genome, and the practicality and affordable cost of analysis using these markers. In this context, aiming to contribute to the economic development and the sustainable management, the objective of this study is to investigate the genetic diversity of wild and cultivated populations of *A. gigas* and the potential of a panel composed by 12 microsatellite markers, combined in a multiplex-PCR system, for the individual and population genetic tracing of this species.

## 2. Materials and Methods

### 2.1. Ethics Statement

This research was approved by the Ethics Committee on Animal Use (CEUA) of the Federal Rural University of Amazon (UFRA), protocol number 055/2017 (CEUA)–23084.017501/2017-02 (UFRA). 

### 2.2. Sample Collection and DNA Extraction 

**Figure 1:**
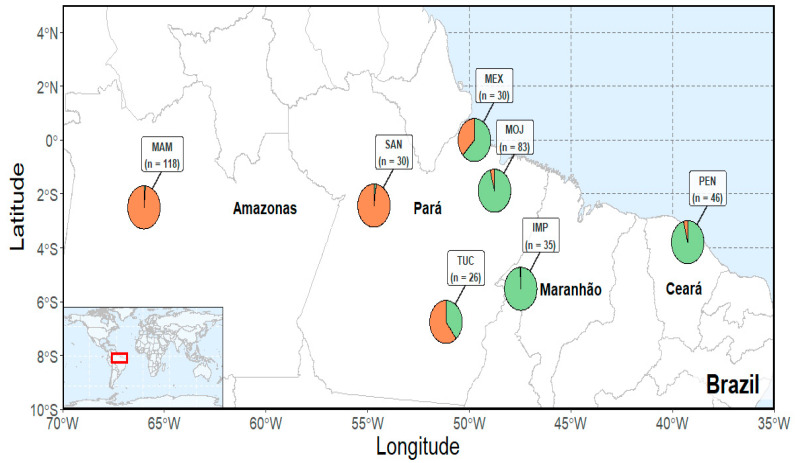
Map of collecting localities of *Arapaima gigas*. Pie plots indicate average population ancestry of each of the three main biological clusters detected in STRUCTURE analysis (see Figure 2 and Figure 3). Color scheme is same as in Figure 2.

We collected 2 g of muscle tissue of 368 specimens of *A. gigas* belonging to seven populations, including farmed and wild ones. One-hundred and ninety of these samples were obtained from four farmed populations located at the state of Pará, in the Northern region of Brazil (Tucumã—TUC and Moju—MOJ), and from the states of Maranhão and Ceará, in the Brazilian Northeast (Imperatriz—IMP and Pentecostes—PEN). The other 178 samples come from three wild populations, located at the states of Pará and Amazonas, in the Northern region of Brazil (Mamiráua—MAM, Santarém—SAN and Mexiana—MEX) (Figure 1).

The samplings were preserved in 70% ethanol (Merck KGaA, Darmstadt, Germany) and posteriorly stored in −20 °C. Total genomic DNA was extracted from the digested tissue in proteinase K solution/Sodium Dodecyl Sulfate (SDS) (Thermo Scientific, Waltham, Massachusetts, United States) and purified in Phenol/Chloroform (Invitrogen, Waltham, Massachusetts, United States), followed by precipitation in Isopropanol (Merck KGaA, Darmstadt, Germany) [12]. The DNA concentration was measured in the NanoDrop ND1000 spectrophotometer (Thermo Scientific, Waltham, Massachusetts, United States).

### 2.3. Amplification and Genotyping of the Microsatellites

Twelve microsatellite loci were analyzed (Agig13519, Agig50571, Agig58115, Agig08356, Agig67103, Agig93614, Agig33291, Agig90836, Agig05001, Agig70664, Agig08912, and Agig06409), developed and selected as the most polymorphic loci for *A. gigas* by [13], and these loci were mixed in a multiplex-PCR system.

The multiplex PCR was run in a final volume of 9.5 µL, using a 5.0 µL 2X Qiagen Multiplex PCR Master Mix (Qiagen, Hilden, Germany), 1.0 µL of Q-Solution (Qiagen), 2.0 µL of H2O, 0.5 µL of primer mix, and 1.0 µL of genomic DNA, described by [13]. Amplification reactions were performed in a Veriti thermocycler (Applied Biosystems, Waltham, Massachusetts, United States). The thermocycling conditions were as follows: initial denaturation at 95 °C for 15 min, followed by 10 cycles at 94 °C for 30 s, 60 °C for 90 s, and 72 °C for 60 s; 20 cycles at 94 °C for 30 s, 58 °C for 90 s, and 72 °C for 60 s, and a final extension at 72 °C for 60 min, 10 °C for 5 min. 

The samples were genotyped in an ABI 3130 Genetic Analyzer (Applied Biosystems Inc., Foster, CA, USA) using a mixture of 1 μL of the PCR product, 8.5 μL of formaldehyde and 0.5 μL of GeneScan 500 LIZ (Applied Biosystems). The determination of fragment size and allele designation was conducted with the GeneMapper 3.7 software (Applied Biosystems).

### 2.4. Data Analysis

#### 2.4.1. Genetic Diversity, Inbreeding and Population Structure Analyses 

The presence of the null allele in the markers was checked using the program Micro-Checker 2.2.3 [14]. The Polymorphic Information Content (PIC) of each marker was calculated in Cervus 3.0 [15], using [16]’s classification system, in which values of less than 0.25 indicate low polymorphism; those of 0.25–0.5, a moderate level of polymorphism, and those higher than 0.5, a highly polymorphic locus.

The genetic diversity indexes, the number of alleles per locus (NA) and the allelic richness (A_R_) were analyzed in Fstat 2.9.3.2 [17]. The observed heterozygosity (H_O_), the expected heterozygosity (H_E_) and Hardy–Weinberg Equilibrium (HWE) was determined in Arlequin 3.5.2.2 [18]. HWE *p*-values were adjusted by Bonferroni correction [19].

The effective population size (Ne) was estimated in COLONY v2.0.6.4 [20] assuming random mating and a 95% parametric confidence interval. The inbreeding coefficient was estimated as F = ½ N_E_.

The genetic differentiation among populations was estimated by pairwise F_ST_ [21] and R_ST_, calculated in Arlequin 3.5 [18], according to the Hartl and Clark classification system [22]. The existence of genetic substructure and admixture between populations wild and farmed was determined using a Bayesian cluster analysis run in Structure 2.3.4 [23], adjusted by the method proposed by Evanno [24]. Besides, we also examined population structure using a model of multivariate ordination approach for discriminant analysis of principal components (DAPC), using the Adegenet package in R [25].

#### 2.4.2. Individual and Population Genetic Tracin

The capacity for the tracing of the *A. gigas* was evaluated for both individual and population identification methods. This analysis was based on 1000 genotypes from each population (total of 7000 genotypes), simulated by P-Loci [26] based on the first 10 pairs sampled at seven populations. In this simulation, sex was identified alternately as male or female.

The efficacy of the tracing by the identification of individuals was evaluated using the methods full likelihood, run in COLONY v2.0.6.4 [20]. To identify the population from each individual, we used the Bayesian method of Rannala and Mountain in Geneclass 2.0 [27]. The probability of assigning a specimen to a given population was calculated using the maximum likelihood estimation and expressed by scores.

The performance of each method used to allocate the specimens to their respective populations was evaluated based on the criteria adopted by Larrain et al., 2014 [28], i.e., sensitivity (S), calculated by the number of individuals assigned correctly to their respective population divided by the total number of individuals in this population; specificity (E), that is, the number of individuals correctly excluded from the population divided by the number of individuals that should be excluded from the population, and the average probability assignment score (AP), calculated by the mean likelihood of each successful identification for each population.

## 3. Results

### 3.1. Genetic Diversity, Inbreeding and Population Structure

The results of the Micro-Checker analysis indicated that there were null alleles in the markers Agig93614, Agig90836, Agig70664, Agig08912, and Agig06409. The 12-microsatellite system used in this research presented extremely informative PIC average rates for the MAM, SAN, MEX, and TUC populations. PIC moderate rates were observed for the populations of PEN, IMP, and MOJ (Supplementary Table S1).

The indices of genetic variability for each of the 12 microsatellites analyzed for each population are shown in complement Table S1. The 12-loci microsatellite system identified 102 alleles in the seven analyzed populations. The most polymorphic locus was the Agig90836, which presented 13 alleles, while the least polymorphic one was the Agig08912, presenting 5 alleles. 

The average N_A_ value varied from 2.83 to 7.08 and the average A_R_ varied from 2.79 to 5.99. (Table 1). Wild populations of MAM (N_A_ = 7.08, A_R_ = 5.86) and SAN (N_A_ = 6.08, A_R_ = 5.99) presented high rates of genetic diversity, in contrast to the farmed population of IMP, which presented the lowest rates (N_A_ = 2.83, A_R_ = 2.79). The remaining populations presented intermediate N_A_ and A_R_ rates (Table 1).

The population of SAN was the only one in Hardy–Weinberg Equilibrium. The other populations (MAM, MEX, TUC, PEN, IMP, and MOJ) presented at least one marker in disequilibrium, either for deficiency and/or heterozygosity (Supplementary Table S1). The lowest values of H_O_ and H_E_ were observed for the populations of MOJ (H_O_ = 0.47) and PEN (H_E_ = 0.51), respectively, while the highest values were observed in IMP (H_O_ = 0.71) and TUC (H_E_ = 0.69) (Table 1).

All farmed populations (TUC, PEN, IMP, and MOJ) presented a drastic reduction in N_E_, and endogamic depletion by F statistics. The wild population of MAM presented a higher value of N_E_ = 168 and a low inbreeding rate, F = 0.003. Critical records of N_E_ and F rates were observed for the farmed populations of IMP, N_E_ = 4 and F = 0.125 (Table 2). 

**Figure 2:**
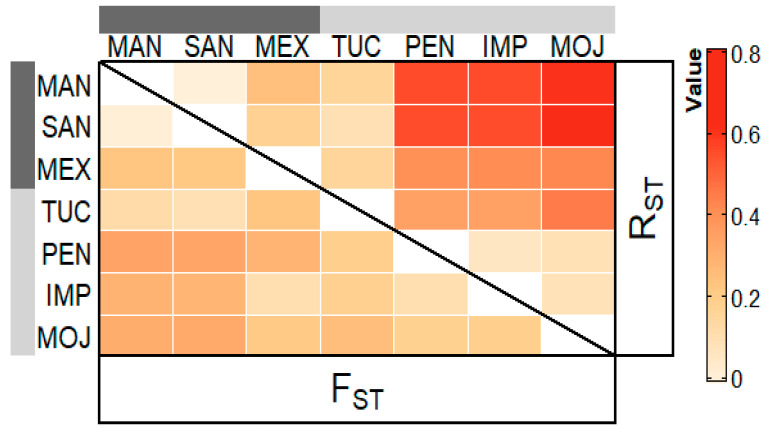
Heat map of the pairwise F_ST_ values (above the diagonal) and the pairwise R_ST_ values (below the diagonal) for the three wild and four farmed populations of *Arapaima gigas*, estimated by microsatellite data.

R_ST_ and F_ST_ rates above 0.150 revealed high genetic differences among the farmed populations of PEN, IMP, and MOJ when compared to the wild populations of MAM, SAN and MEX. R_ST_ and F_ST_ rates varying from 0 to 0.050 indicate low differences between MAM and SAN populations, and that both populations are highly different from the MEX one. The comparison among the farmed populations (PEN × IMP × MOJ), revealed F_ST_ and R_ST_ rates varying from 0.050 to 0.150, indicating that they have a moderate differentiation. The population from TUC presented high differences when compared to the populations from MAM, MEX, PEN, IMP, and MOJ, and moderate difference when compared to SAN (Figure 2).

**Figure 3:**
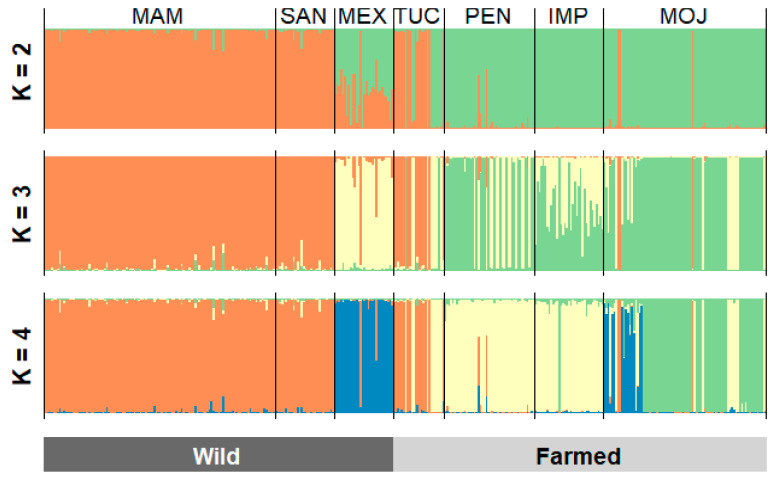
Standard population structure of three wild and four farmed populations of *Arapaima gigas*. The analysis based on 12-loci microsatellite markers, supported by STRUCTURE, indicated the existence of two groups (K = 2). MAM—Mamirauá; SAN—Santarém; MEX—Mexiana; TUC—Tucumã; PEN—Pentecostes; IMP—Imperatriz; MOJ—Moju.

The Bayesian clustering analysis in STRUCTURE was based on the criteria proposed by [12] and indicated that the seven populations are divided into two different subpopulations (K = 2). The first cluster is formed by the populations MAM, SAN, MEX and TUC. The second cluster is composed of the populations from PEN, IMP, and MOJ. It was not possible to analyze substructure in K = 3 and K = 4 (Figure 3 and Supplementary Figure S2).

DAPC genetic structure analysis reveals that the seven populations are divided into four subpopulations. The first cluster is composed of the wild populations of MAN and SAN. The second group is formed by the farmed populations of IMP, MOJ, and PEN. The population from TUC formed the third cluster, which was genetically closer to the first and second clusters. The fourth group was composed of the wild population from MEX and was genetically more distant from the other ones.

### 3.2. Individual and Population Genetic Tracing

Individual identification based on the Full Likelihood method was able to correctly identify 100% of the simulated genotypes in populations from SAN and MOJ, by both parental probabilities (maternal and paternal). The other populations, MAM, MEX, TUC, IMP and PEN, had more than 90% of their genotype identified (Table 3).

The identification at the population level, using the Bayesian method was able to distinguish 100% of the simulated genotypes for the populations of MEX, TUC and MOJ. The analysis for the populations of MAM, SAN, PEN, and IMP was able to correctly identify more than 99% of the genotypes. Maximum specificity was observed for all populations and maximum sensibility was seen for the populations of MAM, SAN, MEX, TUC, and MOJ. A lower sensibility was observed for the populations of PEN and IMP (Table 4).

## 4. Discussion

### 4.1. Genetic Diversity, Inbreeding and Population Structure

The Amazon basin is home to the largest biodiversity of freshwater fish in the world, there are about 2300 recognized species, corresponding to more than 15% all of the freshwater fish species described on the planet [29], not counting the new ones that are described annually. However, all this aquatic biodiversity is under strong threat due to deforestation, construction of dams and navigable waterways, pollution and overfishing [30,31]. It is believed that anthropogenic impacts have a much greater effect on fish fauna in the Amazon than any predicted climate change [32]. Among the most endangered species, the *A. gigas* is one of the main sources of food, fishing and trade in riverside communities in the Amazon, and more recently has aroused the interests of aquaculture farming.

Unfortunately, since 1975 *A. gigas* has been on the IUCN list of endangered species, within the “data deficient” category, meaning there is a lack of data to assess its population status. Thus, tools such as the multiplex microsatellite panel described by [13] are critical for obtaining genetic data and trade monitoring. In the present study, this panel was applied in the analysis of wild and farmed populations of the *A. gigas*, being efficient in answering questions on genetic diversity and tracing. The PIC values of the 12 combined markers allowed the obtention of accurate data t for statistical analysis.

The seven populations of *A. gigas* investigated in this study showed low rates of genetic diversity, N_A_, A_R_, H_O_, H_E_, PIC, N_E_ and F—especially in the N_A_ and A_R_ rates (Table 1), compared to the *Colossoma macropomum* [33], an Amazonian species that, like the *A. gigas*, has great economic importance for fishing and aquaculture. Several studies, such as the ones performed by several authors before [34,35,36,37,38,39,40,41], also studied the genetic diversity of wild stocks of the *A. gigas* in different locations throughout the Amazon basin, presenting different results that range from low to high genetic variability.

A common point among the populations is the record of higher rates of genetic diversity in populations belonging to the Mamirauá reserve, thus reinforcing the importance of the participatory management model used in this region.

Our results warn the loss of genetic diversity in the farmed populations from TUC, IMP, PEN, and MOJ, when compared to the wild ones from MAM, SAN, and MEX (Table 1). We assume that the loss of genetic variation is due to genetic bottlenecks, caused by the domestication process and the founding effect. We reinforce that without sufficient genetic variability, there is always a risk that the population will not be able to respond well to new selective pressures caused by environmental changes [42]. We highlight that in farmed populations, this is an issue for the genetic management.

To decrease the high level of inbreeding recorded in the farmed populations of the *A. gigas* (Table 2), measures such as the implantation of reproductive genetic management and recruiting additional founders, aiming to increase the genetic variability and minimize inbreeding, must be adopted.

As in all farmed populations, the reduction of N_E_ is observed in the wild populations of MEX (considering the 50 as the minimum N_E_) [43], meaning they deserve more attention from the competent organizations and entities. This index is seen by conservationists as a measure of the "genetic status" of a population [44], which helps in understanding the genetic health, evaluating the risk of inbreeding and inbreeding depression, thus, also the risk of extinction [45]. For wild populations, we advise the inclusion of NE with the census size for monitoring the effectiveness of species protection actions. 

We believe that actions commonly used in fish farming environments, such as consanguineous mating and the low number of breeders, contributed to the high genetic differentiation of the farmed populations from PEN, IMP, and MOJ in comparison to the wild ones from MAM, SAN and MEX (Figure 3). The intense employment of these practices added to the length of time of activity, and the founding effect may justify the moderate differentiation registered among the cultivated populations.

The low differentiation between the wild populations of SAN and MAM (Figure 3), supported by the structure pattern revealed by STRUCUTURE and DAPC analysis (Figure 1 and Figure 2), may reflect a historical gene flow between these areas, even though *A. gigas* is considered a sedentary species in the literature. Ref. [34], upon finding high gene flow rates among populations of the *A. gigas* that were up to 1500 km distant, pointed out the important role for juvenile dispersal as a means of conveying gene flow between subpopulations, requiring further studies.

**Figure 4:**
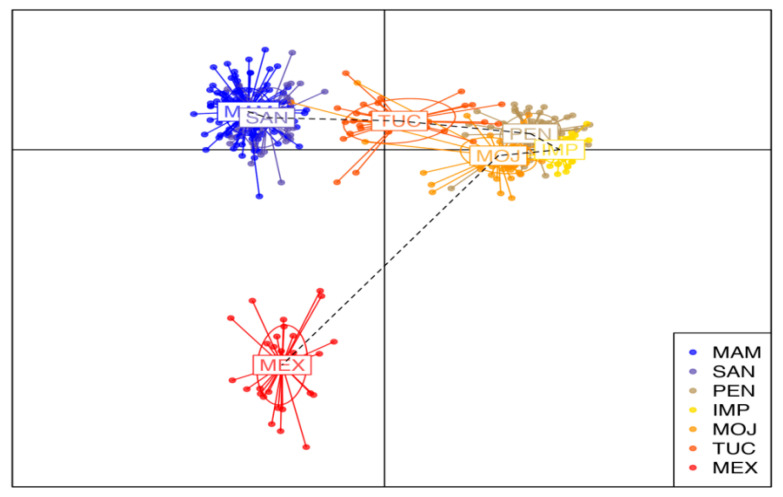
Scatterplot of Discriminant Analysis of Principle Components (DAPC) of three wild and four farmed populations of *Arapaima gigas*. Clusters are shown by different colors and inertia ellipses, while dots represent individuals. Wild population: MAM—Mamirauá; SAN—Santarém; MEX—Mexiana. Farmed population: TUC—Tucumã; PEN—Pentecostes; IMP—Imperatriz; MOJ—Moju.

The high differentiation of the wild population of MEX from the MAM and SAN populations (Figure 3) and the structuring analysis by DAPC (Figure 4) reveal a lower gene flow among these wild populations. This is perhaps indicative of a possible process of isolation by distance, due to environmental factors hindering the high rates of migration, and also distinct anthropogenic factors associated with overexploitation [39].

The formation of two clusters (K = 2) in the Bayesian analysis reinforces the loss of genetic diversity in the farmed populations from PEN, IMP, and MOJ, which is reflected in the pattern of distribution of allele frequencies of these populations in a different cluster apart from the wild populations and also from the population from TUC (Figure 1 and Figure 4). We believe the population from TUC, despite being a farmed population, was structured with the wild ones for containing a high representation of wild individuals in its base population.

When analyzing the structuring of wild populations of the *A. gigas* along the Amazon basin, [34] conclude it is possible to observe changes in the structure of the MEX population, although we have only one species different from what was described by Stewart, 2003 [46]. Our data corroborate these findings (Figure 1), but it is possible to observe changes in the structure of the MEX population (Figure 2 and Figure 4).

In view of the low genetic diversity of the wild populations of *A. gigas*, we reiterate the importance of measures to control the *A. gigas* fishing in the state of Amazonas. We also emphasize the need for the authorities responsible for fisheries management of developing a national standard for species management throughout the entire Amazon basin.

### 4.2. Individual and Population Genetic Tracing

Food traceability is becoming increasingly important for ensuring international food-safety and assisting in the control and sustainability of commercial activities. For this, tools based on DNA analysis have been highlighted, as they present many advantages for DNA being found in all cells—requiring a small amount of material for analysis, and the possibility of the collection at any stage of the production chain [47]. 

The individual and population discrimination tests used for species tracing can be used in different contexts. For instance, in conservation circumstances, they can be used to assess the genetic impact of domesticated specimens on wild populations. Moreover, the genetic tracing can be used in the combat against illegal fishing through the identification of the origin of seized materials, among others. In aquaculture, these tests are commonly used to track farm fleeting, create genetic relationships maps among breeders, and to identify the farm of origin of contaminated stocks, in cases of detection of diseases or toxins in commercialized fish.

For the *A. gigas*, identification at the population level had better performance than the individual discrimination, which corroborates the findings of [11,28]. A simulation study reported that at least 15 microsatellites are necessary to correctly reach 95% of confidence in attribution decisions [48]. Based on the populations used in this study, we demonstrated that with 12 microsatellites our results were more than 99% accurate in correctly identifying the genotype of seven populations, at the population level. Furthermore, at the individual level, only one population presented results under 95%. However, the number of markers required for the genetic tracing is different for each species, and it depends on various factors, such as the genetic diversity and population structure [49].

The 12-loci microsatellite panel tested for the *A. gigas* comes to meet the growing need for reliable, fast and low-cost molecular tools for direct application to improving legal aspects of fish trading, such as the discrimination of farmed individuals from wild ones in/from different locations. For further studies, we recommend an increase in the number of markers on the tested panel, aiming to reach greater accuracy in the analyses, especially in populations that have a lower genetic diversity.

## 5. Conclusions

Genetic tracing using the 12 microsatellite markers was effective and presented a better performance in identifying samples at the population level. The 12-microsatellite panel is appliable in the legal aspects of the trade of the *A. gigas*, such as origin discrimination, reproductive genetic management by DNA profiling, and evaluation and monitoring of genetic diversity. 

## Figures and Tables

**Table 1:** Genetic diversity estimated by 12 microsatellite loci for three wild and four farmed populations of the *A. gigas*.

Average	Population						
	MAM (118)	SAN (30)	MEX (30)	TUC (26)	PEN (46)	IMP (35)	MOJ (83)
N_A_	7.08	6.08	4.67	5.00	5.08	2.83	5.83
A_R_	5.86	5.99	4.59	5.00	4.64	2.79	4.50
H_O_	**0.54**	0.59	**0.50**	**0.64**	**0.55**	**0.71**	**0.47**
H_E_	0.62	0.64	0.57	0.69	0.51	0.52	0.52

N_A_—number of alleles; A_R_—allelic richness; H_E_—expected heterozygosity; H_O_—observed heterozygosity. Wild population: MAM—Mamirauá; SAN–Santarém; MEX—Mexiana. Farmed population: TUC—Tucumã; PEN—Pentecostes; IMP—Imperatriz; MOJ—Moju. Values in bold font—populations presenting markers that are not in Hardy–Weinberg Equilibrium.

**Table 2:** Sample size (N), effective population size (Ne), Confidence Interval (CI) and inbreeding coefficient (F) for the seven populations of the *Arapaima gigas* analyzed in the present study, based on 12 microsatellite loci.

Population	N	N_e_	95% CL	F = 1/2N_e_
MAM	118	168	128–217	0.003
SAN	30	87	52–168	0.006
MEX	30	36	22–68	0.014
TUC	26	11	6–26	0.045
PEN	46	15	9–30	0.033
IMP	35	4	2–12	0.125
MOJ	83	11	6–26	0.045

Wild population: MAM—Mamirauá; SAN—Santarém; MEX—Mexiana. Farmed population: TUC—Tucumã; PEN—Pentecostes; IMP—Imperatriz; MOJ—Moju.

**Table 3:** Individual identification of simulated genotypes of *Arapaima gigas* from three wild and four farmed populations.

Population	N	Full Likelihood Method	
		Maternal Probability	Paternal Probability
MAM	1000	998 (0.904)	998(0.825)
SAN	1000	1000 (0.999)	1000 (0.996)
MEX	1000	995 (0.654)	955 (0.657)
TUC	1000	986 (0.601)	986 (0.336)
PEN	1000	999 (0.621)	999 (0.625)
IMP	1000	917 (0.543)	917 (0.546)
MOJ	1000	1000 (1)	1000 (1)

Wild population: MAM—Mamirauá; SAN—Santarém; MEX—Mexiana. Farmed population: TUC—Tucumã; PEN—Pentecostes; IMP—Imperatriz; MOJ—Moju.

**Table 4:** Performance of identification of the simulated genotypes of *Arapaima gigas* from three wild and four farmed populations.

Populations	Method Bayesian [27]		
	Sensitivity	Specificity	Average Probability Assignment Score
MAM	1	1	0.999
SAN	1	1	0.999
MEX	1	1	1
TUC	1	1	1
PEN	0.997	1	0.998
IMP	0.998	1	0.997
MOJ	1	1	1
Average	0.999	1	0.999

Wild population: MAM—Mamirauá; SAN—Santarém; MEX—Mexiana. Farmed population: TUC—Tucumã; PEN—Pentecostes; IMP—Imperatriz; MOJ—Moju.

## Data Availability

Not applicable.

## Supplementary Materials

The following are available online at https://www.mdpi.com/article/10.3390/genes12091324/s1, Figure S1: Delta K values for STRUCTURE analysis of seven populations of the *Arapaima gigas*—Delta K, calculated according to Evanno et al. [12], is plotted against the number of modeled gene pools (K). Table S1: Genetic diversity estimated by 12 microsatellite loci for three populations wild of the *A. gigas* and four farmed.
