# Supplementary material for: Novel Microsatellite Markers Used for Determining Genetic Diversity and Tracing of Wild and Farmed Populations of the Amazonian Giant Fish Arapaima gigas
Source: Genes (Basel). 2021 Aug 27;12(9):1324. doi: 10.3390/genes12091324 (PMC8467478; doi:10.3390/genes12091324)

Supplementary Materials:

Supplementary Table S1: Genetic diversity estimated by 12 microsatellite loci for three populations wild of the *A. gigas* and four farmed.

Population	Loci												
	Agig13519	Agig50571	Agig58115	Agig08356	Agig67103	Agig93614	Agig33291	Agig90836	Agig05001	Agig70664	Agig08912	Agig06409	Average
						**Mamiráua (118)**							
**NA**	8	11	4	5	7	5	9	13	6	7	4	6	7.083
**AR**	5.907	9.646	3.984	4.219	4.81	4.956	8.518	9.716	4.546	5.344	3.314	5.392	5.863
**HO**	0.66102	0.79661	0.55556	0.37288	0.5339	**0.51724**	0.80342	0.74359	0.25424	0.64103	0.38462	**0.26724**	0.544
**HE**	0.67923	0.84702	0.56608	0.41028	0.54583	0.68783	0.84927	0.85063	0.24158	0.73292	0.50941	0.47529	0.616
**PIC**	0.623	0.826	0.518	0.383	0.493	0.644	0.828	0.829	0.233	0.684	0.413	0.446	0.577
						**Santarém (30)**							
**NA**	9	10	5	5	5	6	8	10	2	4	4	5	6.083
**AR**	8.7	9.833	4.865	4.998	4.732	5.867	7.998	9.949	1.998	4	3.983	4.984	5.992
**HO**	0.66667	0.66667	0.5	0.6	0.63333	0.7	0.86667	0.83333	0.1	0.5	0.5	0.53333	0.592
**HE**	0.78192	0.84859	0.48588	0.5887	0.56271	0.69774	0.84237	0.85424	0.09661	0.65028	0.58475	0.6548	0.637
**PIC**	0.736	0.815	0.446	0.547	0.472	0.651	0.808	0.822	0.09	0.58	0.484	0.6	0.588
**Mexiana (30)**													
**NA**	5	3	6	4	5	6	4	5	5	5	5	3	4.667
**AR**	5	3	5.999	3.733	5	5.925	3.867	4.866	4.984	4.849	4.853	3	4.590
**HO**	**0.4**	**0.16667**	0.72414	0.43333	0.81481	**0.2963**	0.73333	**0.7**	0.63333	**0.3**	**0.46429**	**0.37037**	0.503
**HE**	0.71582	0.27062	0.77495	0.44576	0.73375	0.51852	0.54124	0.63898	0.72712	0.53277	0.53571	0.4689	0.575
**PIC**	0.654	0.25	0.731	0.368	0.673	0.467	0.465	0.557	0.667	0.463	0.448	0.415	0.513
						**Tucumã (26)**							
**NA**	5	5	4	6	4	5	7	7	5	4	4	4	5
**AR**	5	5	4	6	4	5	7	7	5	4	4	4	5
**HO**	**0.462**	0.577	**0.423**	**0.962**	**0.615**	0.731	0.962	**0.846**	0.577	**0.577**	0.423	0.538	0.641
**HE**	0.807	0.719	0.624	0.771	0.612	0.744	0.706	0.857	0.621	0.729	0.413	0.688	0.691
**PIC**	0.758	0.662	0.560	0.725	0.554	0.684	0.661	0.820	0.536	0.666	0.367	0.613	0.634
						**Pentecostes (46)**							
**NA**	4	6	5	5	6	5	7	6	5	5	3	4	5.083
**AR**	3.567	5.719	3.980	4.914	5.796	4.506	5.682	5.378	4.781	4.614	2.995	3.800	4.644
**HO**	0.405	0.565	0.489	0.444	**0.911**	**0.556**	0.761	0.587	0.761	0.478	0.326	0.326	0.551
**HE**	0.386	0.544	0.417	0.432	0.777	0.655	0.596	0.546	0.664	0.415	0.350	0.324	0.509

**PIC**	0.338	0.506	0.358	0.407	0.730	0.579	0.550	0.510	0.597	0.380	0.316	0.300	0.464
**Imperatriz (35)**													
**NA**	3	3	2	4	3	3	3	2	4	2	2	3	2.833
**AR**	3	3	2	3.999	3	3	2.743	2	4	2	2	2.743	2.790
**HO**	0.629	**0.800**	0.657	0.429	0.886	0.829	**1.000**	0.257	**0.943**	0.514	**0.971**	0.629	0.712
**HE**	0.623	0.666	0.448	0.381	0.666	0.655	0.521	0.227	0.653	0.388	0.507	0.498	0.519
**PIC**	0.543	0.582	0.344	0.555	0.582	0.569	0.396	0.199	0.597	0.309	0.375	0.384	0.453
**Moju (83)**													
**NA**	5	8	5	7	7	3	9	7	6	5	3	5	5.833
**AR**	3.843	5.181	3.630	5.373	4.469	2.985	6.477	5.104	5.237	4.907	2.997	3.755	4.497
**HO**	**0.663**	**0.277**	**0.122**	**0.892**	**0.470**	**0.205**	**0.951**	**0.084**	**0.854**	**0.284**	**0.325**	0.512	0.470
**HE**	0.664	0.462	0.213	0.699	0.622	0.287	0.709	0.509	0.675	0.460	0.456	0.542	0.525
**PIC**	0.590	0.432	0.203	0.636	0.550	0.266	0.656	0.469	0.612	0.435	0.403	0.443	0.475

N_A - number of alleles; A_R - allelic richness; H_E - expected heterozygosity; H_O - observed heterozygosity. Wild population: MAM - Mamirauá; SAN - Santarém; MEX Mexiana. Farmed population: TUC – Tucumã; PEN – Pentecostes; IMP – Imperatriz; MOJ – Moju. *Values in bold font – populations presenting markers that are not in Hardy-Weinberg Equilibrium

Supplementary Figure S1. Delta K values for STRUCTURE analysis of seven populations of the *Arapaima gigas*—Delta K, calculated according to Evanno et al. [12], is plotted against the number of modeled gene pools (K).

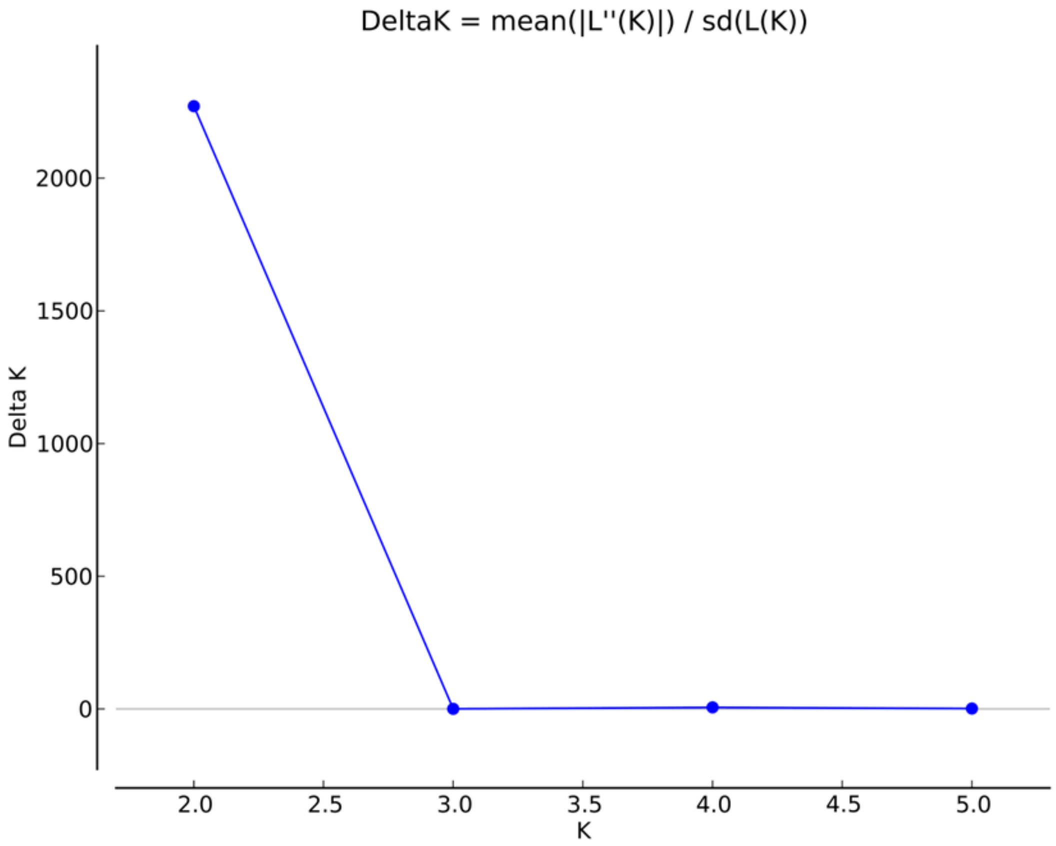

Supplement: Supplementary file 1 [file genes-12-01324-s001.zip › Supplementary Materials.pdf]
