# Supplementary figures and images for: Novel Microsatellite Markers Used for Determining Genetic Diversity and Tracing of Wild and Farmed Populations of the Amazonian Giant Fish Arapaima gigas
Source: Genes (Basel). 2021 Aug 27;12(9):1324. doi: 10.3390/genes12091324 (PMC8467478; doi:10.3390/genes12091324)

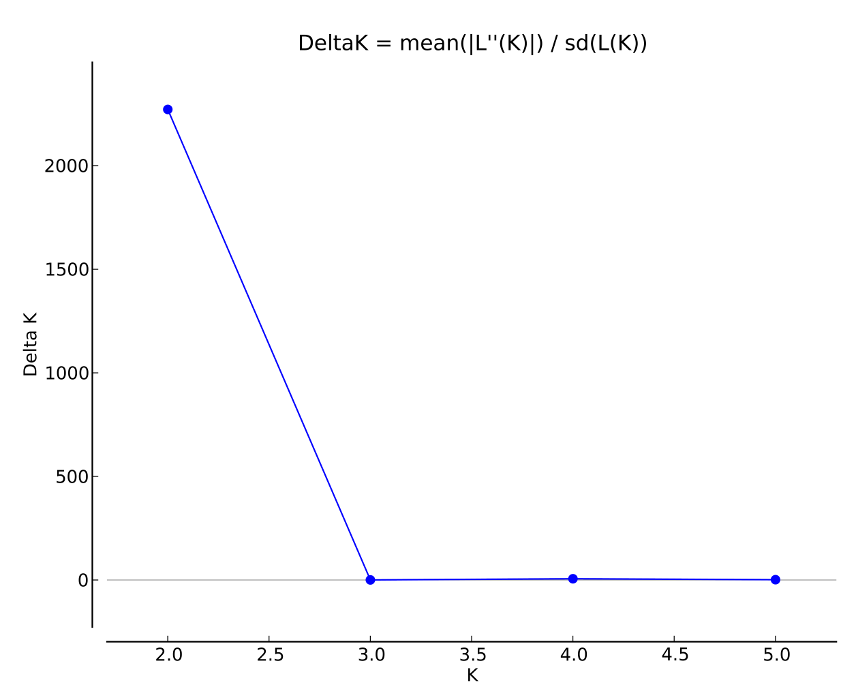

Supplement: Supplementary file 1 [file genes-12-01324-s001.zip › Supplementary Image 1.png]
